# Potential for *in vivo* visualization of intracellular pH gradient in the brain using PET imaging

**DOI:** 10.1093/braincomms/fcae172

**Published:** 2024-05-22

**Authors:** Tomoteru Yamasaki, Wakana Mori, Takayuki Ohkubo, Atsuto Hiraishi, Yiding Zhang, Yusuke Kurihara, Nobuki Nengaki, Hideaki Tashima, Masayuki Fujinaga, Ming-Rong Zhang

**Affiliations:** Department of Advanced Nuclear Medicine Sciences, Institute for Quantum Medical Science, National Institutes for Quantum Science and Technology, Chiba 263-8555, Japan; Department of Advanced Nuclear Medicine Sciences, Institute for Quantum Medical Science, National Institutes for Quantum Science and Technology, Chiba 263-8555, Japan; Department of Advanced Nuclear Medicine Sciences, Institute for Quantum Medical Science, National Institutes for Quantum Science and Technology, Chiba 263-8555, Japan; SHI Accelerator Service Co. Ltd., Tokyo 141-0031, Japan; Department of Advanced Nuclear Medicine Sciences, Institute for Quantum Medical Science, National Institutes for Quantum Science and Technology, Chiba 263-8555, Japan; Department of Advanced Nuclear Medicine Sciences, Institute for Quantum Medical Science, National Institutes for Quantum Science and Technology, Chiba 263-8555, Japan; Department of Advanced Nuclear Medicine Sciences, Institute for Quantum Medical Science, National Institutes for Quantum Science and Technology, Chiba 263-8555, Japan; SHI Accelerator Service Co. Ltd., Tokyo 141-0031, Japan; Department of Advanced Nuclear Medicine Sciences, Institute for Quantum Medical Science, National Institutes for Quantum Science and Technology, Chiba 263-8555, Japan; SHI Accelerator Service Co. Ltd., Tokyo 141-0031, Japan; Department of Advanced Nuclear Medicine Sciences, Institute for Quantum Medical Science, National Institutes for Quantum Science and Technology, Chiba 263-8555, Japan; Department of Advanced Nuclear Medicine Sciences, Institute for Quantum Medical Science, National Institutes for Quantum Science and Technology, Chiba 263-8555, Japan; Department of Advanced Nuclear Medicine Sciences, Institute for Quantum Medical Science, National Institutes for Quantum Science and Technology, Chiba 263-8555, Japan

**Keywords:** hydrolysis, hypoxia, intracellular pH, monoacylglycerol lipase, PET

## Abstract

Intracellular pH is a valuable index for predicting neuronal damage and injury. However, no PET probe is currently available for monitoring intracellular pH *in vivo*. In this study, we developed a new approach for visualizing the hydrolysis rate of monoacylglycerol lipase, which is widely distributed in neurons and astrocytes throughout the brain. This approach uses PET with the new radioprobe [^11^C]QST-0837 (1,1,1,3,3,3-hexafluoropropan-2-yl-3-(1-phenyl-1*H*-pyrazol-3-yl)azetidine-1-[^11^C]carboxylate), a covalent inhibitor containing an azetidine carbamate skeleton for monoacylglycerol lipase. The uptake and residence of this new radioprobe depends on the intracellular pH gradient, and we evaluated this with *in silico*, *in vitro* and *in vivo* assessments. Molecular dynamics simulations predicted that because the azetidine carbamate moiety is close to that of water molecules, the compound containing azetidine carbamate would be more easily hydrolyzed following binding to monoacylglycerol lipase than would its analogue containing a piperidine carbamate skeleton. Interestingly, it was difficult for monoacylglycerol lipase to hydrolyze the azetidine carbamate compound under weakly acidic (pH 6) conditions because of a change in the interactions with water molecules on the carbamate moiety of their complex. Subsequently, an *in vitro* assessment using rat brain homogenate to confirm the molecular dynamics simulation-predicted behaviour of the azetidine carbamate compound showed that [^11^C]QST-0837 reacted with monoacylglycerol lipase to yield an [^11^C]complex, which was hydrolyzed to liberate ^11^CO_2_ as a final product. Additionally, the ^11^CO_2_ liberation rate was slower at lower pH. Finally, to indicate the feasibility of estimating how the hydrolysis rate depends on intracellular pH *in vivo*, we performed a PET study with [^11^C]QST-0837 using ischaemic rats. In our proposed *in vivo* compartment model, the clearance rate of radioactivity from the brain reflected the rate of [^11^C]QST-0837 hydrolysis (clearance through the production of ^11^CO_2_) in the brain, which was lower in a remarkably hypoxic area than in the contralateral region. In conclusion, we indicated the potential for visualization of the intracellular pH gradient in the brain using PET imaging, although some limitations remain. This approach should permit further elucidation of the pathological mechanisms involved under acidic conditions in multiple CNS disorders.

## Teaser

Yamasaki *et al*. report the development of [^11^C]QST-0837 as a novel PET ligand for quantification of monoacylglycerol lipase activity and its potential for *in vivo* visualization of intracellular pH gradient in the brain using PET imaging.

## Graphical Abstract

**Graphical Abstract:**
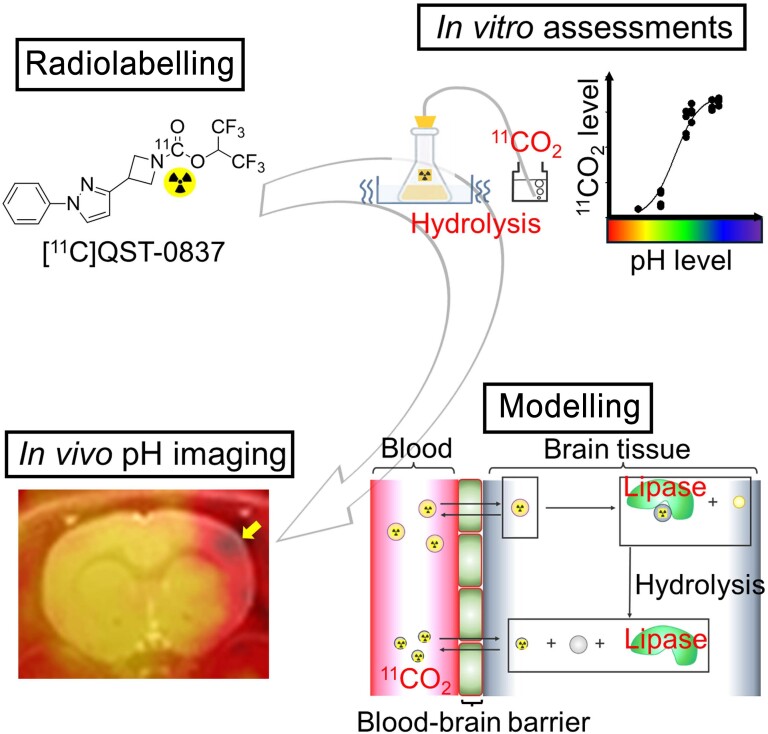

## Introduction

Cerebral vascular hypoxia damages the CNS^1–4^ by switching aerobic energy metabolism to anaerobic metabolism, resulting in intracellular hyperaccumulation of acidic residues such as protons, lactate and carbonic acid.^5^ Subsequently, the neuronal synapse is depolarized by the disproportion of cations (Na^+^ and K^+^) because the production of ATP decreases under anaerobic metabolism. The intracellular Ca^2+^ concentration then increases and excess neurotransmitters (e.g. glutamate) are released, inducing neurotoxicity, neuroinflammation and neuronal death.^6,7^ Such acute damage can lead to a number of pathologies such as depression, post-stroke dementia and, potentially, neurodegeneration.^3^ Therefore, monitoring changes in intracellular pH (pHi), which depend on the intracellular accumulation of acidic sources, should be very important for predicting neuronal damage in the early stage of several CNS disorders.

PET is an advanced imaging technique that is widely used in basic and clinical studies to elucidate the kinetics, molecular density and distribution of drugs *in vivo*. To date, only two major radioprobes have been developed for pH-weighted PET imaging. Four decades ago, [^11^C]dimethyloxazolidinedione ([^11^C]DMO), the membrane permeability of which depends on the pH gradient, was first developed as a pH-weighted marker for PET studies.^8,9^ However, PET imaging with [^11^C]DMO was inaccurate because the biodistribution of [^11^C]DMO could not distinguish between plasmalemmal, intracellular and extracellular pH gradients.^2^ Two decades later, [^64^Cu]DOTA-pHLIP, which specifically binds to the membranes of low-pH cells, was identified.^10^ However, the binding of [^64^Cu]DOTA-pHLIP depends on the extracellular pH gradient, but not on the pHi. To the best of our knowledge, no pHi-sensitive PET probe is currently available for use in humans.

**Figure 1:**
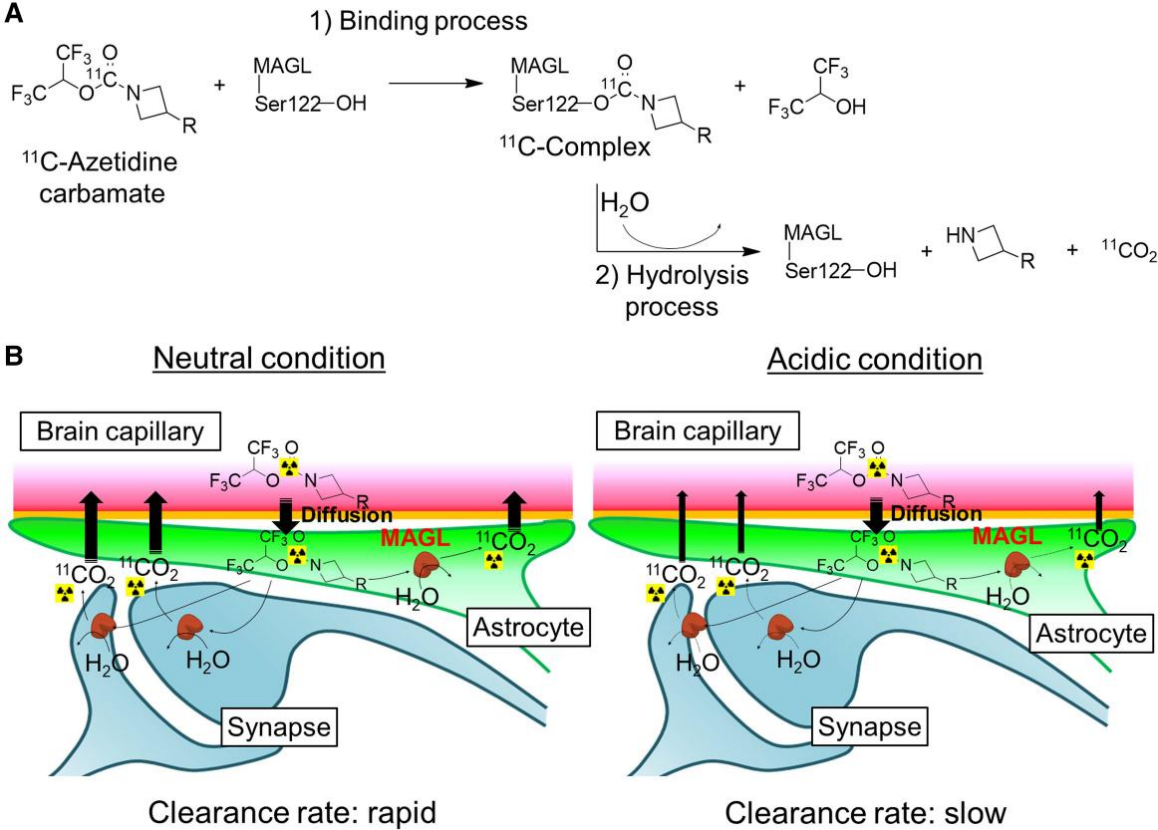
**The hypothesized mechanism for hydrolysis of the ^11^C-labelled MAGL inhibitor containing an azetidine carbamate skeleton**. (**A**) Proposed disassociation route for the ^11^C-labelled MAGL inhibitor containing an azetidine carbamate moiety. (**B**) Diagram illustrating the effect of acidic sources on ^11^CO_2_ production.

Recently, Butler *et al*.^11^ reported azetidine and piperidine carbamates as covalent inhibitors of monoacylglycerol lipase (MAGL), an enzyme widely distributed throughout the mammalian brain and a key enzyme for regulation of the endocannabinoid system. Our group recently developed radiolabelled azetidine carbamate inhibitors for use as PET probes for imaging of MAGL (Supplementary Fig. 1A). Although they were designed as irreversible PET probes, they showed unexpected rapid radioactivity clearance after a high initial uptake of radioactivity in the brain, similar to that shown by reversible PET probes.^12–14^ Such brain kinetics strongly suggest that carbamate inhibitors covalently bind to MAGL, resulting in unstable complexes in the brain. In this study, following information in a previous report,^15^ we hypothesized a disassociation mechanism (Fig. 1A) for [^11^C]azetidine carbamates in the brain. [^11^C]Azetidine carbamate covalently reacts with MAGL to form an [^11^C]complex, which is subsequently hydrolyzed, resulting in ^11^CO_2_ as a final radioactive product. Because ^11^CO_2_ is rapidly cleared from the brain,^16^ we considered that the radioactivity clearance rate would depend mainly on the rate of hydrolysis of the [^11^C]complex within the brain.

Enzyme activity is reduced at pH values outside the optimal range, generally because changes in pH affect enzyme–substrate complex formation, substrate ionization and the 3D structure of enzymes. More importantly, MAGL is widely expressed throughout the brain and is intracellularly localized not only in pre- and post-synaptic neurons, but also in astrocytes.^17,18^ Therefore, estimation of the ^11^CO_2_ production rate through hydrolysis of the [^11^C]complex formed between azetidine and MAGL may permit monitoring of regional changes in pHi within the brain (Fig. 1B).

Following on from this insight, we developed 1,1,1,3,3,3-hexafluoropropan-2-yl-3-(1-phenyl-1*H*-pyrazol-3-yl)azetidine-1-[^11^C]carboxylate ([^11^C]QST-0837; IC_50_ = 0.2 nM) as a novel radioprobe for visualizing the pHi gradient. For comparison with [^11^C]QST-0837, we simultaneously synthesized its piperidine analogue (1,1,1,3,3,3-hexafluoropropan-2-yl-4-(1-phenyl-1*H*-pyrazol-3-yl)piperidine-1-[^11^C]carboxylate; [^11^C]HPPC; IC_50_ = 2.0 nM). We first performed molecular dynamics (MD) simulations for the complexes between azetidine carbamate or piperidine carbamate compounds and MAGL and then confirmed their structural relationship with *in vivo* hydrolytic instability. Subsequently, we radiosynthesized [^11^C]QST-0837 and [^11^C]HPPC and tested their ability to measure the pHi gradient *in vitro* and *in vivo*.

## Materials and methods

### General

All chemical reagents and organic solvents were purchased from Sigma–Aldrich (St. Louis, MO, USA), FUJIFILM Wako Pure Chemical Industries (Osaka, Japan), or Nacalai Tesque (Kyoto, Japan) and were used without further purification. ^11^C was produced using a cyclotron (CYPRIS HM-18; Sumitomo Heavy Industries, Tokyo, Japan).

### Animals

Male Sprague–Dawley (SD) rats (*n* = 24, 7–10 weeks old) weighing 280–350 g were purchased from Japan SLC (Shizuoka, Japan), kept in a temperature- and humidity-controlled environment with a 12-h light–dark cycle and allowed to freely consume a standard diet (MB-1/Funabashi Farm, Chiba, Japan) and water. All animal experiments were performed according to the recommendations specified by the Committee for the Care and Use of Laboratory Animals of the National Institutes for Quantum Science and Technology (QST) and Animal Research and the Reporting of In Vivo Experiments (ARRIVE) guidelines and were approved by the Institutional Committee of QST (approval number: 16-1006).

### MD simulations

A 3D MAGL structure (PDB code: 6AX1), including the ligand-binding domain, was used for modelling and MD simulations. The initial structures of the carbamate inhibitor (MAGL complexes) (complex-A for azetidine carbamate and complex-P for piperidine carbamate) were constructed based on model ligands (compound-A for azetidine carbamate and compound-P for piperidine carbamate) in the homology module of the molecular modelling software MOE (Chemical Computing Group, Montreal, Canada). MD simulations for complex-A were conducted under neutral (pH 7) and weakly acidic (pH 6) conditions. Under neutral conditions, Asp and Glu residues and the C-terminus of MAGL were kept deprotonated (COO^−^, Lys and Arg residues), the N-terminus of the protein was kept protonated and the His residues of MAGL were kept deprotonated. Under weakly acidic conditions, the His residues of MAGL were protonated.

The complex-A and complex-P were solvated in a truncated octahedral TIP3P water box with a thickness of 8 Å around the complex. Then, protein.ff14SB, water.tip3p and a general amber force field were used as force fields. The systems were neutralized by adding Na^+^ ions as the counter ions. The energy minimized (MM) calculations and MD simulations were performed using AMBER18/PMEMD with periodic boundary conditions of constant temperature (300 K) and pressure (1 atm) and a non-bonded interaction cut-off distance of 8 Å. The particle mesh Ewald method was employed to calculate the electrostatic interactions. After tethering the heavy atoms of the protein, the MM calculations of the system were performed in 1000 steps using the steepest descent and conjugated gradient methods. MD simulations were performed only for water molecules in the system within a 50 ps time frame, whilst increasing the temperature from 0 to 300 K. Subsequently, the C*α* atoms of the protein were tethered, and then, MM calculations of the side chain of the protein were performed in 1000 steps using the steepest descent method and in 10 000 steps using the conjugated gradient method. MD calculations for the C*α* atoms of the protein were performed under the same conditions as described above. MM calculations for the system were performed in 1000 steps using the steepest descent method and in 10 000 steps using the conjugated gradient method. Finally, the MM calculations and MD simulations were performed for the entire system. Atoms were heated from 0 to 300 K at 50 ps intervals, and the whole system reached the equilibrium state for MD simulation (5 ns) of the complex structures under constant temperature (300 K) and pressure (1 atm). For each complex, structural changes were evaluated using the root mean square deviation (RMSD) of the trajectory of the intermediate structures in the MD simulations.

### Radiochemistry

^11^CO_2_ was produced via ^14^N(p, *α*)^11^C nuclear reactions using a cyclotron (Cypris HM18, Sumitomo Heavy Industries) and was transferred into a pre-heated mechanizer packed with a nickel catalyst at 400°C to produce ^11^CH_4_, which was subsequently reacted with chlorine gas at 560°C to generate ^11^CCl_4_. ^11^COCl_2_ was produced via the reaction of ^11^CCl_4_ with iodine oxide and sulphuric acid and was trapped in a solution of 1,1,1,3,3,3-hexafluoropropan-2-ol (3.1 μl) and 1,2,2,6,6-pentamethylpiperidine (PMP; 5.4 μl) in tetrahydrofuran (200 µl) at 0°C.^19^ The solution was then heated to 30°C for 3 min. A solution of precursors of [^11^C]QST-0837 or [^11^C]HPPC (1.00 mg, see Supplementary Fig. 2) with PMP (2.3 µl) in tetrahydrofuran (200 µl) was added to the mixture and heated at 30°C for 3 min, and the solvent was then removed at 80°C before cooling to room temperature (r.t.) (Supplementary Fig. 3A). After dilution with 1 ml of separation solvent, the mixture was purified by high-performance liquid chromatography (HPLC; CAPCELL Pak C18 column; 10 mm i.d. × 250 mm, 5 µm) using a mobile phase of CH_3_CN/H_2_O/Et_3_N (70/30/0.1, v/v/v, for [^11^C]QST-0837) or CH_3_CN/H_2_O/TFA (75/25/0.1, v/v/v, for [^11^C]HPPC) at a flow rate of 5.0 ml/min. The respective retention times were 11.0 min for [^11^C]QST-0837 and 11.7 min for [^11^C]HPPC. The HPLC fractions of [^11^C]QST-0837 and [^11^C]HPPC were collected in a flask containing 25% ascorbic acid in sterile water (100 µl), and Tween 80 (75 µl) in ethanol (0.3 ml) was added before synthesis and evaporation to dryness. The final product was collected in a sterile flask and reformulated in sterile normal saline (2 ml). The resulting compound was analysed using HPLC with a UV detector of 254-nm wavelength and a radioactivity detector. The radiochemical and chemical purities were measured by analytical HPLC (CAPCELL Pak C18 column; 4.6 mm i.d. × 250 mm, 5 µm) as a mobile phase of CH_3_CN/H_2_O/Et_3_N (70/30/0.1, v/v/v, for [^11^C]QST-0837) or CH_3_CN/H_2_O/TFA (75/25/0.1, v/v/v, for [^11^C]HPPC), at a flow rate of 1.0 ml/min. The identification of [^11^C]QST-0837 and [^11^C]HPPC was confirmed by co-injection with compound-A or compound-P, respectively, and the retention times in radio-HPLC analyses were 10.3 min for [^11^C]QST-0837 and 10.8 min for [^11^C]HPPC. The calculated lipophilicities (cLogP) of each radioligand were estimated using ChemBioDraw Ultra (version 12.0; PerkinElmer, Waltham, MA, USA).

### *In vitro*  ^11^CO_2_ collection assay

Six rats were anesthetized with isoflurane (1–5% in air) and exsanguinated. The brain was quickly removed and homogenized with three equivalents of saline using a homogenizer (Silent Crasher S; Heidolph, Schwabach, Germany). The homogenate (6 ml) was added to a conical flask containing saline (2 ml) and lactic acid (1 ml) at various concentrations (0, 10, 30, 50 and 100 mM). The flask was closed with a rubber plug through which were inserted glass tubes to allow bubbling. An *in vitro*  ^11^CO_2_ collection assay was performed according to a previous report with modification.^20^ Briefly, [^11^C]QST-0837 (1 ml, 10–18 MBq) or [^11^C]HPPC (1 ml, 10–11 MBq) was added to the flask and it was incubated in a 37°C water bath with shaking for 60 min. During the incubation, a tube was inserted into the bottle containing 10 ml of 2-aminoethanol (50% in distilled water). The air for bubbling was then automatically browsed using an air pump connected to another tube. The bottle was then changed for a new one after 5 min. Before stopping the enzymatic reaction, the pH of the brain homogenates was measured using a pH metre (Elite pH Spear Pocket Testers; Thermo Fisher Scientific, Waltham, MA, USA). Subsequently, pre-chilled CH_3_CN (10 ml) was quickly added to the flask to stop the enzymatic reaction and the mixture was separated into several micro-tubes. The micro-tubes were centrifuged at 10 000 *g*, and the supernatants were transferred into new micro-tubes. An autogamma scintillation counter (2480 WIZARD^2^; PerkinElmer) was used to measure the radioactivity levels of the bottle for collecting ^11^CO_2_, the pellet and the supernatant. Radioactivity is expressed as a percentage of the incubation dose (%ID).

### Permanent middle cerebral artery occlusion surgery

The permanent middle cerebral artery occlusion (MCAO) model was established in 10 rats. The rats were anesthetized with isoflurane (1.5% in air) and their body temperature was maintained at approximately 37°C using a heating pad (BWT-100; Bio Research Center Co., Ltd., Aichi, Japan). The MCAO surgery was performed using the Koizumi method.^21^ Briefly, using a surgical microscope (CLS 150MR; Leica Microsystems, Wetzlar, Germany), a silicon-coated monofilament (Doccol Corporation, Sharon, MA, USA) was introduced into the internal carotid artery via a cut in the external carotid artery and was passed until the monofilament occluded the MCAO base. After 1, 3–4, or 6 h of occlusion, the rats were used for the PET imaging study.

### Small-animal PET imaging

Before PET assessment, the rats were anesthetized with isoflurane (5% in air) and a 24-gauge intravenous catheter (Terumo Medical Products, Tokyo, Japan) was inserted into their tail vein. For the blocking study, compound-A (unlabelled QST-0837), compound-P (unlabelled HPPC) and JW642 (MedChemExpress, Monmouth Junction, NJ, USA) in different doses (1 or 3 mg/kg) were administered via the tail vein catheter 30 min before the injection of the radioprobe.^12,13^ Subsequently, the rats were secured in a custom-designed chamber and placed in a small-animal PET scanner (Inveon; Siemens Medical Solutions, Knoxville, TN, USA). Their body temperatures were maintained using a 40°C water circulation system (T/Pump TP401; Gaymar Industries, Orchard Park, NY, USA). A bolus of [^11^C]QST-0837 (1 ml, 52–57 MBq, 0.3–0.9 nmol) or [^11^C]HPPC (1 ml, 41–60 MBq, 1.0–1.2 nmol) was injected at a flow rate of 0.5 ml/min via the tail vein catheter. For the chase study, JW642 at 1 mg/kg was administered via the tail vein catheter 20 min after a bolus injection of radioprobe. Dynamic emission scanning in 3D list mode was performed for 90 min (1 min × 4 frames, 2 min × 8 frames and 5 min × 14 frames). The acquired dynamic PET images were reconstructed by filtered back-projection using a Hanning filter with a Nyquist cut-off of 0.5 cycles/pixel. The time–activity curves (TACs) of [^11^C]QST-0837 and [^11^C]HPPC were manually acquired for volumes of interest (VOIs) in the cerebral cortex, striatum (caudate/putamen), hippocampus, thalamus, pons and cerebellum, by referring to a rat brain MRI template in PMOD software (version 3.4; PMOD technology, Zurich, Switzerland). For the MCAO model, VOIs were manually drawn on both the ipsilateral and contralateral sides of the brain by visually assigning the affected regions. The radioactivity was decay-corrected to the injection time and is expressed as the standardized uptake value (SUV).^22^ The rate of hydrolysis (*K*_H_) was estimated by a one-exponential fitting on the TACs from 15–90 min, as follows:


(1) $$\mathrm{Radioactivity}(t)={\mathrm{Ae}}^{\wedge }\left(-\mathrm{at}\right),$$

(2) $$a=K_{H}.$$

*In vivo* mapping scaled with the relative *K*_H_ value was generated as follows:

1. Frames from 3 to 10 (2–15 min) in PET dynamic images are averaged, which are regarded as the apparent maximum uptake of radioactivity.
2. Frames from 11 to 26 (15–90 min) in PET dynamic images are averaged.
3. Image for total volume of the efflux of radioactivity from the brain can be reconstructed by subtracting (2) from (1).
4. Images scaled with relative *K*_H_ value are obtained by dividing (3) by (1).

All procedures were conducted using fusion tool of PMOD software.

### 2,3,5-Triphenyl tetrazolium chloride staining

After PET scanning, the rats were euthanized by cervical dislocation. The brain was quickly removed, placed on a brain slicer (EMJapan Co., Ltd., Tokyo, Japan) and cut into 2-mm coronal sections. The sections were incubated in 2% 2,3,5-triphenyl tetrazolium chloride (TTC, dissolved in phosphate-buffered saline) for 20 min at r.t.

### Immunofluorescent staining for hypoxia

Tissue hypoxia levels were assessed using Hypoxyprobe™ (Hypoxyprobe Inc., Burlington, MA, USA) immunofluorescence staining.^23^ Briefly, after MCAO rats were occluded for 4 h, Hypoxyprobe™ (60 mg/kg body weight) was injected intraperitoneally. The rats were sacrificed after a PET scan (90 min) and their brains were quickly removed and frozen on powdered dry ice. Coronal brain sections were cut at −20°C using a cryostat (NX50; Thermo Fisher Scientific, Waltham, MA, USA) and mounted on MAS-coated glass slides (Matsunami-glass, Osaka, Japan). Cryo-sections were prepared with a 20 μm thickness and a 100 μm distance between two adjacent sections. For the assay, brain sections were fixed in 4% paraformaldehyde/PBS for 15 min at r.t. and then blocked with blocking serum for 20 min at r.t. The sections were incubated with anti-hypoxyprobe mouse IgG as the primary antibody for 30 min at r.t. After thorough washing, the sections were incubated with a biotinylated secondary antibody (1:100) for 30 min at r.t., followed by fluorescent staining using a TSA fluorescence system (PerkinElmer). The slides were then mounted with medium (Vector Laboratories, Burlingame, CA, USA). The fluorescent signals and areas with fluorescent staining in regions of interest (ROIs) shown in Supplementary Fig. 4 were quantified using ImageJ software (http://rsbweb.nih.gov/ij/). The fluorescence intensities were compared with *K*_H_ values estimated on TACs acquired from VOIs on brain slices adjacent from ROIs drawn as Supplementary Fig. 4 in the same individual in the PET study using [^11^C]QST-0837.

### Statistical analysis

Statistical analyses were performed using GraphPad Prism (version 5.0; GraphPad Software, San Diego, CA, USA). Differences between means were assessed as appropriate, using two-way ANOVA followed by Bonferroni *post hoc* analysis.

## Results

### MD simulations

**Figure 2:**
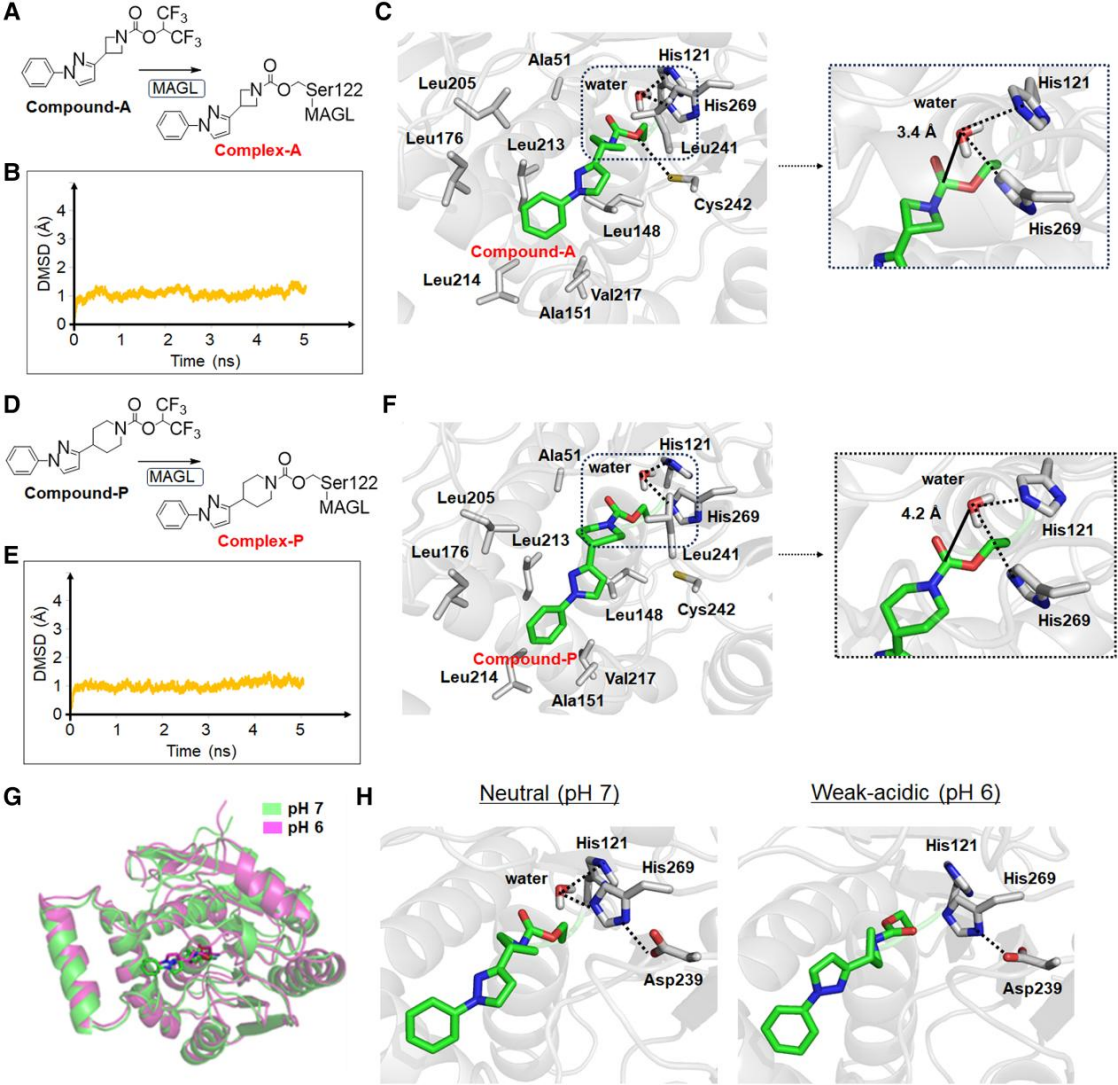
**MD simulations**. (**A**) Chemical structures of compound-A and its MAGL complex. (**B**) Chart of the RMSD values for main chain atoms of complex-A in the MD simulations (5 ns) under neutral conditions. (**C**) 3D structures of complex-A. (**D**) Chemical structures of compound-P and its MAGL complex. (**E**) Chart of RMSD values for main chain atoms of complex-P in the MD simulations. (**F**) 3D structures of complex-P. (**G**) Overlaid structures of complex-A composed from MD simulations at pH 7 and pH 6. (**H**) Changes in the interaction of complex-A with water molecules between neutral (pH 7) and weakly acidic (pH 6) conditions. Spatial localization and interaction of the residue of complex-A with a water molecule at pH 7 (left). Changes in the interaction between the residue of complex-A and a water molecule at pH 6 (right). Hydrogen bonds in the complexes are shown as dotted lines. MD simulations were performed using 3D structures of MAGL (PDB: 6AX1).

To understand the *in vivo* hydrolytic instability of complex-A, we performed MD simulations under neutral conditions similar to normal physiological conditions and compared the results between complex-A and complex-P (Fig. 2A–F). The RMSD values for complex-A and complex-P showed no significant changes in MD simulations (5 ns) under constant temperature (300 K; Fig. 2B and E). Because water is essential for the enzyme hydrolysis, we further analysed the accessibility of water molecules to the respective complexes. By forming hydrogen bonds, His269 and His121 in MAGL were able to capture the water surrounding the carbonyl moiety in both complexes. However, the water molecule was closer to the carbonyl moiety of complex-A (3.4 Å) than to that of complex-P (4.2 Å). Moreover, a hydrogen bond was formed between complex-A and Cys242 in MAGL, which was absent in the case of complex-P because of the longer distance (5.0 Å). These differences indicated that complex-A was hydrolyzed more easily than complex-P. Moreover, the structural strain of the azetidine skeleton is larger than that of the piperidine skeleton, leading to easier hydrolysis.

As mentioned above, enzyme activity is generally weakened by divergence from the optimum pH. Therefore, in the present study, we also compared the interaction of complex-A with water molecules at pH values between neutral (pH 7) and weakly acidic (pH 6). The RMSD value calculated by overlaying the final structures of the MAGL main chain between pH 6 and pH 7 was 1.9 Å (Fig. 2G), suggesting that the *in vivo* binding affinity of compound-A for MAGL would not change at pH 6. By estimating the distance between the carbonyl moiety and the water molecule, we found that the imidazole ring in His269 of MAGL could form a hydrogen bond with water molecules under neutral conditions (Fig. 2H). However, at pH 6, water molecules would have difficulty accessing the carbonyl moiety because they would not be captured by the imidazole ring in MAGL, thus enhancing the stability of complex-A against hydrolysis. Moreover, His269 in MAGL maintained a strong electronic interaction with Asp239 in MAGL under the weakly acidic condition. This was possibly due to the protonation of the imidazole ring in His269 under such conditions, conditions that would also hinder the hydrolysis of complex-A.

### Radiochemistry

To validate our proposed pHi gradient detection approach shown in Fig. 1B, we radiosynthesized [^11^C]QST-0837 and [^11^C]HPPC according to the methods in our previous report, using ^11^COCl_2_ as a labelling agent.^19^ Starting with 30 GBq of ^11^CO_2_, [^11^C]QST-0837 and [^11^C]HPPC were synthesized with 0.9 ± 0.5 GBq (3.1 ± 1.5%) and 1.1 ± 0.6 GBq (4.3 ± 2.3%) at the end of synthesis (EOS), respectively. The radiochemical purities of [^11^C]QST-0837 and [^11^C]HPPC were >98%, and the molar activities at the EOS were 101 ± 24 GBq/μmol for [^11^C]QST-0837 (*n* = 11) and 62 ± 13 GBq/μmol for [^11^C]HPPC (*n* = 5). No radiolysis was observed up to 90 min after formulation, suggesting the radiochemical stability of [^11^C]QST-0837 and [^11^C]HPPC over the duration of at least one PET scan. General chemical profiles (molecular weight, lipophilicity and IC_50_ value for MAGL) for [^11^C]QST-0837 and [^11^C]HPPC are shown in Supplementary Fig. 3B.

### *In vitro*  ^11^CO_2_ collection assay

**Figure 3:**
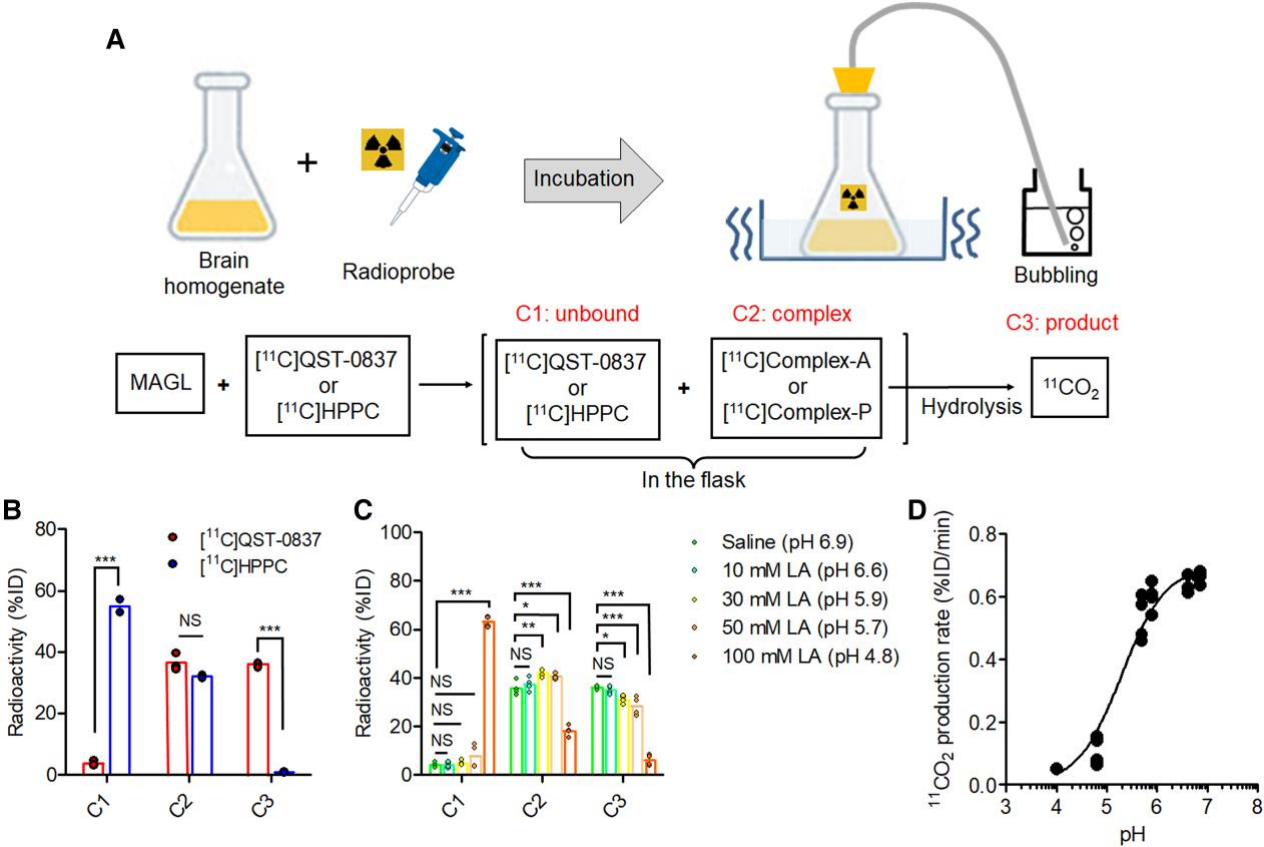
***In vitro*  ^11^CO_2_ collection assay using rat brain homogenate**. (**A**) Schematic illustration and compartments of the assay. (**B**) Radioactivity (percentage of incubation dose, %ID) derived from [^11^C]QST-0837 or [^11^C]HPPC in the three divided compartments (C1: unbound, C2: complex and C3: product). Data from three independent experiments for each radioprobe are shown as mean ± SD. NS: Not significant, ***P < 0.001 (two-way ANOVA with Bonferroni *post hoc* test). (**C**) Radioactivity (%ID) derived from [^11^C]QST-0837 in the presence of different concentrations (0, 10, 30, 50 and 100 mM) of lactic acid (LA). Data from four independent experiments for each LA concentration are shown as mean ± SD. NS: Not significant, *P < 0.05, **P < 0.01 and ***P < 0.001 (two-way ANOVA with Bonferroni *post hoc* test). (**D**) The pH-response curve for ^11^CO_2_ production rate. The pH evoking half the maximal ^11^CO_2_ production was 5.3.

To confirm the production of ^11^CO_2_ resulting from MAGL hydrolysis, we conducted *in vitro*  ^11^CO_2_ collection assessments for [^11^C]QST-0837 and [^11^C]HPPC using rat brain homogenate (Fig. 3A). Figure 3B shows the radioactivity in three divided compartments (C1: unbound radioprobe, C2: complex with MAGL and C3: ^11^CO_2_ as a final product). In the C1 compartment representing the unreacted radioprobe, the radioactivity of [^11^C]QST-0837 was almost eliminated (<5%ID), whereas that of [^11^C]HPPC remained at over 50%ID. The radioactivity in the C2 compartment representing the [^11^C]complex-A or [^11^C]complex-P was approximately 35%ID for both. These results suggest that the reaction between each radioprobe and MAGL reached a saturation dose of approximately 35%ID. More importantly, production of ^11^CO_2_ was detected with [^11^C]QST-0837 (approximately 35%ID), but not with [^11^C]HPPC (<1%ID). This result indicates that [^11^C]complex-A was hydrolyzed under the test conditions, resulting in ^11^CO_2_, whereas [^11^C]complex-P was not hydrolyzed.

We subsequently determined the effect of pH on the rate of ^11^CO_2_ production using an *in vitro*  ^11^CO_2_ collection assay with multiple concentrations of LA. Figure 3C shows the radioactivity generated in each compartment at different pH values. In the C3 compartment, radioactivity gradually decreased with a lowering of pH. The pH evoking half the maximal ^11^CO_2_ production was 5.3 (Fig. 3D). In contrast, the radioactivity in the C2 compartment was approximately 40%ID with all LA concentrations except for 100 mM (pH 4.8), which resulted in a large decrease in radioactivity, similar to that in the C3 compartment. However, the radioactivity in the C1 compartment after treatment with 100 mM LA increased to >60%ID. These results suggest that under severely acidic conditions, the ^11^CO_2_ production rate is slow (<pH 4.8) and the binding affinity of [^11^C]QST-0387 to MAGL is decreased.

### PET imaging in healthy rats

**Figure 4:**
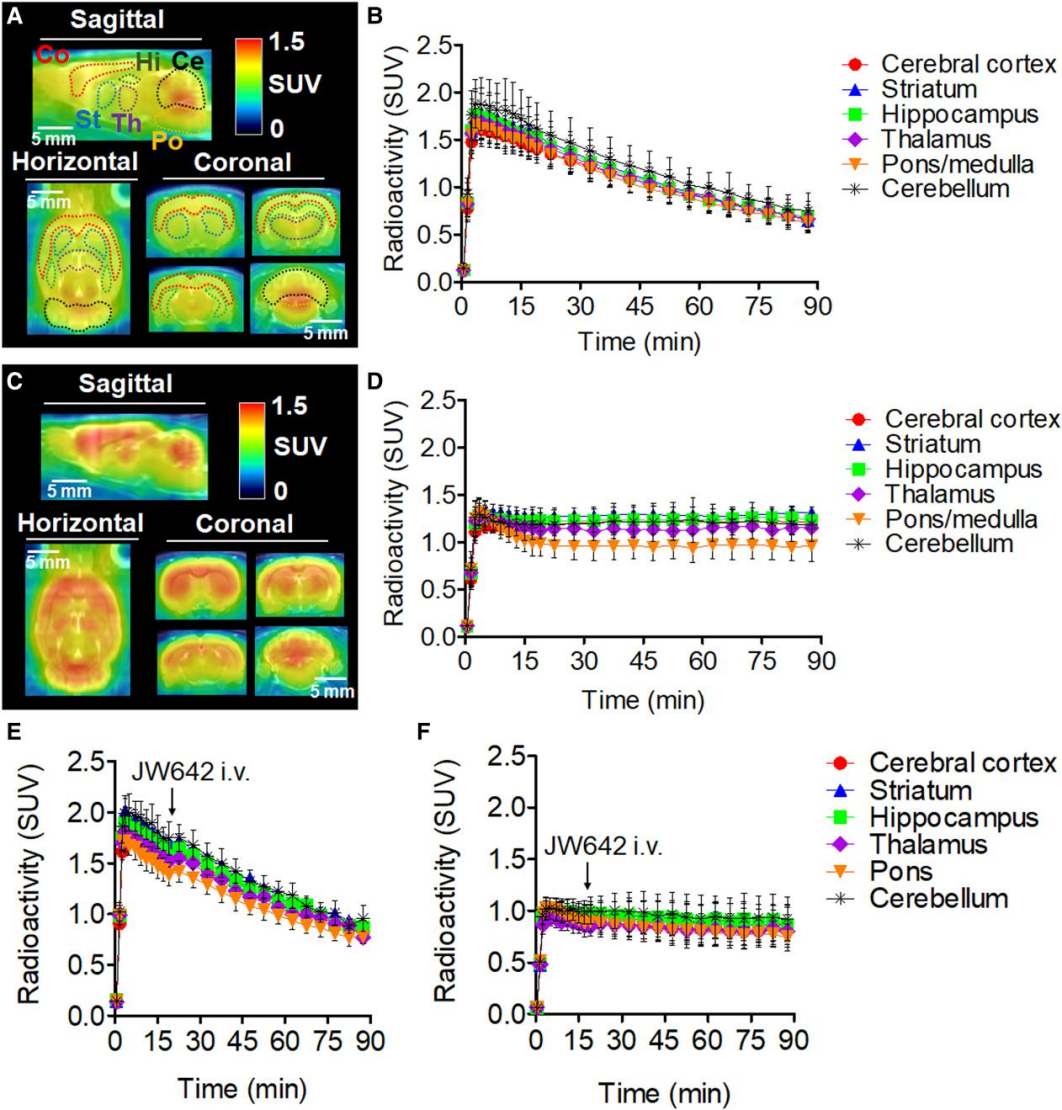
**PET imaging in the brain of a healthy rat**. (**A**) Representative 0–90 min summed PET–MRI images of [^11^C]QST-0837. (**B**) Time–activity curves (*n* = 4) of [^11^C]QST-0837 in the cerebral cortex, striatum, hippocampus, thalamus, pons/medulla and cerebellum. (**C**) Representative 0–90 min summed PET–MRI images of [^11^C]HPPC. (**D**) Time–activity curves (*n* = 4) of [^11^C]HPPC in the cerebral cortex, striatum, hippocampus, thalamus, pons/medulla and cerebellum. (**E** and **F**) Chase studies of [^11^C]QST-0837 and [^11^C]HPPC using an irreversible-type inhibitor for MAGL (JW642). (**E**) Time–activity curves (*n* = 3) of [^11^C]QST-0837 in the brain of rat treated with JW642 (1 mg/kg, i.v.) 20 min after the scan started. (**F**) Time–activity curves (*n* = 3) of [^11^C]HPPC in the brain of rat administered with JW642 (1 mg/kg, i.v.) 20 min after the scan started. ROIs were drawn in the cerebral cortex, striatum, hippocampus, thalamus, pons and cerebellum. Radioactivity is expressed as the SUV. Co, cerebral cortex; St, striatum; Hi, hippocampus; Th, thalamus; Po, pons/medulla; Ce, cerebellum.

Figure 4A–D show representative PET images and TACs of [^11^C]QST-0837 and [^11^C]HPPC across brain regions. PET images of both radioprobes showed widespread radioactive signals throughout the brain. For [^11^C]QST-0837, the radioactivity showed a gradual clearance after the initial uptake. However, [^11^C]complex-P was not hydrolyzed *in vivo* and the radioactivity remained at 1.0–1.4 SUV without clearance at 15 min post-injection. The highest radioactive uptake for [^11^C]QST-0837 was 1.6–1.9 SUV in each investigated region, which was slightly higher than that of our previously developed [^11^C]compound 6 (1.4–1.7 SUV). This was possibly because of the adequate lipophilicity (cLogP = 3.36) and the 2-fold higher affinity of [^11^C]QST-0837 (IC_50_ = 0.2 nM) to MAGL compared with that of [^11^C]compound 6 (IC_50_ = 0.4 nM).^14^

Figure 4E shows chase studies of [^11^C]QST-0837 and [^11^C]HPPC to determine the irreversible binding of these radioprobes for MAGL. In this study, the commercial inhibitor JW642 was used as a potent irreversible inhibitor for MAGL because of its structural similarity. Pre-treatment with 1 mg/kg JW642 (i.v.) significantly decreased brain uptake of both radioprobes, the same as self-blocking (Supplementary Fig. 5). However, there were no changes in the radioactive uptake of either radioprobes when JW642 was post-administered, which strongly suggests that [^11^C]QST-0837 and [^11^C]HPPC are irreversibly bound to MAGL in brain tissue.

### Estimation of hydrolysis rate (*K*_H_) in PET with [^11^C]QST-0837

**Figure 5:**
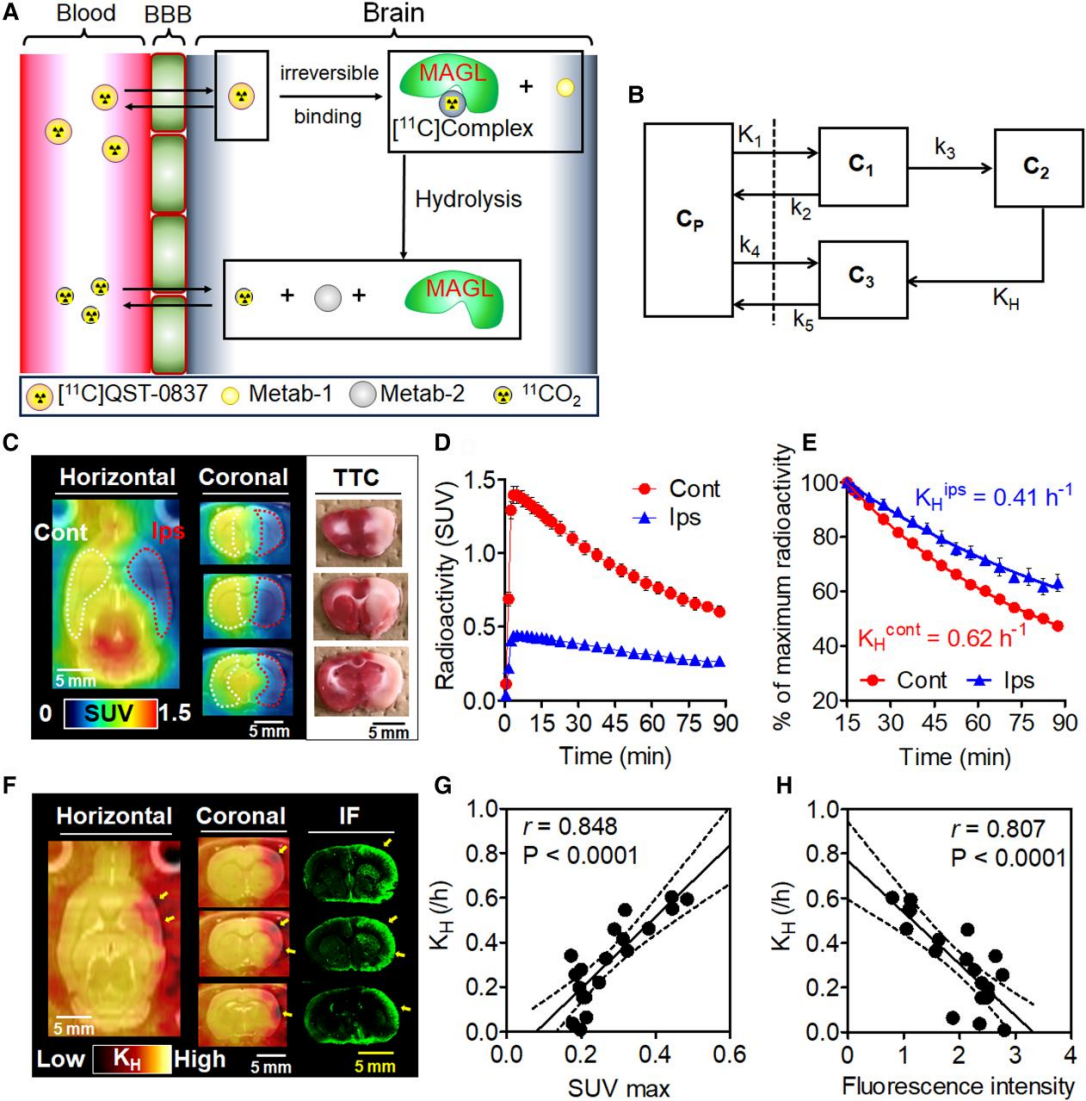
**PET imaging in the brain of a MCAO rat**. (**A**) Diagrammatic reasoning of the clearance of radioactivity starting from [^11^C]QST-0837. (**B**) Schematic of a compartment model for estimation of the kinetic parameters of the radioactivity. C_P_: free radioactive compounds containing [^11^C]QST-0837 and ^11^CO_2_ in the plasma; C1: free and non-specific binding of [^11^C]QST-0837 in the brain tissue; C2: state under [^11^C]complex-A; C3: ^11^CO_2_ as the final product of hydrolysis in the brain tissue; *K*_1_: influx rate of [^11^C]QST-0837; *k*_2_: efflux rate of [^11^C]QST-0837; *k*_3_: association rate of [^11^C]QST-0837 to MAGL; *K*_H_: hydrolysis rate of MAGL; *k*_4_: reuptake rate of ^11^CO_2_; *k*_5_: efflux rate of ^11^CO_2_ from the brain side. (**C**) Representative PET images with [^11^C]QST-0837 and TTC staining in the brains of rats subjected to MCAO for 3–4 h. PET images were generated by summing for 0–90 min after injection and scaling with the SUV. The brain slices were prepared after the PET scan and were stained using TTC. (**D**) Time–activity curves (TACs) of [^11^C]QST-0837 in contralateral (Cont) and ipsilateral (Ips) sides. (**E**) Clearance rate of radioactivity in contralateral (Cont) and ipsilateral (Ips) sides. Radioactivity is expressed as the percentage of maximum radioactivity (SUV of 15 min). *K*_H_ was generated using a mono-exponential fitting. (**F**) PET images scaled with relative *K*_H_ value and immunofluorescent (IF) images reflecting hypoxic regions in the MCAO rat brains. Arrows indicate areas of intense reduction in *K*_H_ value and strong fluorescent signals. (**G**) Correlation plots between the maximum SUV value (SUV max) in TACs acquired from VOIs on brain slice adjacent from ROIs (shown in Supplementary Fig. 4) for quantification of immunohistochemical signals and *K*_H_ values estimated on their TACs. (**H**) Correlation plots between quantitative values for immunohistochemical signal in ROIs (shown in Supplementary Fig. 4) on three slides and *K*_H_ values estimated on TACs obtained from VOIs on brain slice adjacent from these ROIs. Relationship tests were conducted by a linear regression.

Here, we propose a compartment model (Fig. 5A and B) to understand the dynamics of [^11^C]QST-0837 in the brain, based on the results of the PET studies using healthy rats and the predicted transformation of [^11^C]azetidine carbamate shown in Fig. 1A. When [^11^C]QST-0837 is injected, it enters the bloodstream, reaches the brain capillaries (C_P_), passively crosses (*K*_1_) the blood–brain barrier (BBB) and enters brain tissue (C1). Subsequently, [^11^C]QST-0837 irreversibly binds (*k*_3_) to intracellular MAGL to form [^11^C]complex-A (C2 compartment), which gradually hydrolyzes (*K*_H_), producing ^11^CO_2_ (C3 compartment), and the radioactivity from ^11^CO_2_ is rapidly cleared (*k*_5_) from the brain tissue. Theoretically, when the input function of the radioactivity is close to a negligible value (e.g. 15 min after the injection), the clearance rate of radioactivity in the TAC would depend on *K*_H_ and the efflux rate (*k*_5_) of ^11^CO_2_. The efflux rate of ^11^CO_2_ has been reported to be 1.74 min^−1^ in the brains of healthy humans,^16^ which is very rapid. Therefore, in our PET experiment, we considered the clearance rate of the radioactivity on PET with [^11^C]QST-0837 to be the *K*_H_ value. Here, the clearance rate of the radioactivity is simply estimated by a mono-exponential fitting with a non-linear least squares regression.^24^ In the brain of healthy rat, the radioactivity clearance rates (≈*K*_H_) were 0.66 ± 0.06 h^−1^ for the cerebral cortex, 0.75 ± 0.05 h^−1^ for the striatum, 0.71 ± 0.05 h^−1^ for the hippocampus, 0.71 ± 0.05 h^−1^ for the thalamus, 0.72 ± 0.07 h^−1^ for the pons/medulla and 0.69 ± 0.08 h^−1^ for the cerebellum. Interestingly, although differences of initial radioactive uptakes were observed in individuals and brain regions, the clearance rate was the similar level in all subjects and investigated brain regions. Additionally, there was no relationship between estimated maximum radioactive uptake and clearance rate (Supplementary Fig. 6).

### PET imaging for measurement of pHi in the brains of experimental stroke model rats

Figure 5C and D show representative averaged PET images of [^11^C]QST-0837 and TACs for the ipsilateral and contralateral sides of the brains of MCAO rats subjected to 3–4-h occlusion. Radioactive signals disappeared from the ipsilateral side of the forebrain, including the cortex, striatum and amygdala, which appeared as light (white) areas after TTC staining. Additionally, the initial radioactive uptake in the ipsilateral area was approximately 30% of that in the contralateral side. This indicates that the cerebral blood flow (CBF) on the ipsilateral side dropped by 70%. Figure 5E shows a plot of the radioactive clearance in the ipsilateral and contralateral sides of the brains of MCAO rats, including the results of mono-exponential regression analyses. The *K*_H_ values of [^11^C]complex-A in the ipsilateral and contralateral sides were 0.41 and 0.62 h^−1^, respectively. In MCAO rats with 3–4 h of occlusion, the *K*_H_ value in the ipsilateral area was 34% lower than that in the contralateral area. Furthermore, the *K*_H_ values of rats with 1 and 6 h of occlusion were 0.53 and 0.49 h^−1^, respectively, in the ipsilateral sides and 0.66 and 0.63 h^−1^ in the contralateral sides. The *K*_H_ value on the ipsilateral side was 25% lower than that on the contralateral region at 1 h after occlusion and 17% lower at 6 h after occlusion (Table 1). These results correspond with previously reported values for time-dependent changes in intracellular lactate levels in the MCAO model.^25–27^

**Table 1:** Hydrolysis rate (*K*_H_) of MAGL in pMCAO rats

*K*_H_ (h^−1^)	Occlusion time (h)		
	1 ^a^	3–4 ^b^	6 ^a^
Contralateral region	0.62 ± 0.07	0.62 ± 0.05	0.67 ± 0.07
Ipsilateral region	0.47 ± 0.10*	0.41 ± 0.07***	0.56 ± 0.11
Ips/Cont ratio	0.75 ± 0.10	0.66 ± 0.07	0.83 ± 0.10

^a^ *n* = 3. ^b^  *n* = 4. *P < 0.05, ***P < 0.001 (contralateral versus ipsilateral).

The lower *K*_H_ value reflects anaerobic glycolysis and intracellular acidosis, and therefore, we further investigated heterogeneous *K*_H_ values during hypoxia in the brains of MCAO rats at 3–4-h occlusion. Moreover, we compared the *K*_H_ values in brain regions with immunofluorescent signals corresponding to hypoxia. As shown in Fig. 5F, low *K*_H_ values were detected in the upper lip region of the primary somatosensory cortex, secondary somatosensory cortex, insular cortex and piriform cortex, all of which correspond to regions that suffer a severe reduction in CBF following MCAO.^28^ In these regions, the maximum SUV value was also low and positively correlated with the *K*_H_ value (Fig. 5G). Moreover, immunofluorescent signals on slides corresponding to ROIs in the PET images showed a strong negative correlation (*r* > 0.8, *P* < 0.0001) with *K*_H_ values (Fig. 5H).

## Discussion

In this study, we developed ^11^COCl_2_-labelled azetidine carbamate ([^11^C]QST-0837), which targets MGAL. Then, utilizing the discovery that [^11^C]QST-0837 is slowly hydrolyzed by MAGL, we successfully demonstrated *in vivo* mapping of pHi according to the hydrolytic activity of MAGL in the brains of MCAO rats.

To investigate why the radiolabelled covalent MAGL inhibitors containing an azetidine carbamate skeleton (developed as an irreversible-type PET probe) showed unexpected clearance of radioactivity from the brain in the PET study, we performed MD simulations using a 3D MAGL structure and compound-A (Fig. 2). These MD simulations showed that the Cys242 of MAGL formed a hydrogen bond with the azetidine carbamate, but not with piperidine carbamate (Fig. 2C and F). Thus, we suggest that the difference in the interaction with MAGL between azetidine and piperidine carbamates is caused by structural differences in the carbon ring and the degree hydrophobicity. The formation of a hydrogen bond between azetidine carbamate and Cys242 could fix a spatial arrangement of the azetidine carbamate moiety that would be easily hydrolyzed by MAGL because of the increased accessibility of the water molecule. Further MD simulations suggested that complex-A would be structurally stable under weakly acidic conditions (pH 6) because the accessibility of the carbonyl moiety to water molecules would be hindered. MAGL is classified into the serine protease family, which structurally includes Ser122, His269 and Asp239 as the catalytic triad, three amino acids important for enzyme activity.^29,30^ Since in MD simulations the difference in the RMSD values on the main chain of complex-A between pH 7 and pH 6 was 1.9 Å, the main chain of the 3D structure of MAGL indicated no obvious structural changes at pH 6. However, under a weakly acidic condition such as pH 6, the His269 of the catalytic triad and other histidines in MAGL were protonated, resulting in formation of an ionic bond between His269 and Asp239 (Fig. 2H). We suggest that these reactions shifted the spatial arrangement of the side chain of His269 in MAGL, and that consequently, MAGL lost its function as a catalytic triad to trap the water molecule around the carbonyl moiety. Thus, the hydrolyzation of complex-A could depend on pH conditions, and therefore, estimation of the rate of hydrolysis of [^11^C]QST-0837 by MAGL could be used as an indicator of *in vivo* pHi.

As shown in Fig. 1A, we hypothesized that azetidine carbamate [^11^C]QST-0837 was bound to MAGL in the brain and hydrolyzed, resulting in production of ^11^CO_2_. Therefore, following successful ^11^C-labelling using ^11^COCl_2_ as a radiolabelled intermediate, we conducted *in vitro*  ^11^CO_2_ collection assays using rat brain homogenate for [^11^C]QST-0837 and piperidine carbamate [^11^C]HPPC (as a comparator). In these assays, the radioactivity could be present in three compartments, which represent the unbound condition (unreacted), the complex and the production of ^11^CO_2_. As expected, radioactivity in the ^11^CO_2_ compartment was significantly detected with [^11^C]QST-0837, but not with [^11^C]HPPC. These results strongly support the modelled differences (in the MD simulations) in the structural resistance of compound-A and compound-P to hydrolysis by MAGL. Furthermore, for [^11^C]QST-0837, we investigated the change in ^11^CO_2_ production rate according to the change in pH. As predicted in the MD simulations, the production rate of ^11^CO_2_ decreased at lower pH values. Although highly acidic conditions (<pH 4.8) seemed to deactivate MAGL by denaturing it, the binding affinity of [^11^C]QST-0837 to MAGL and the ^11^CO_2_ production were maintained down to pH 5.7. It is known that the optimum pH for MAGL activity in the brain is in the range of 8–9,^31^ whereas the pHi is kept at around 7 in the healthy brain. Therefore, since the pH evoking half the maximal ^11^CO_2_ production was 5.3, this approach could be used to detect activity across a pH range of 5–7.

Prior to our work on the *in vivo* visualization of the pHi gradient using the disease model, we compared the brain kinetics of [^11^C]QST-0837 and [^11^C]HPPC using PET imaging in healthy rats (Fig. 4). [^11^C]QST-0837 containing an azetidine carbamate skeleton showed a moderate clearance of radioactivity after initial uptake, which was not seen with [^11^C]HPPC containing a piperidine carbamate skeleton. However, administration of JW642 (a potent covalent inhibitor of MAGL) after the injection of [^11^C]QST-0837 did not result in changes in radioactivity in the brain regions showing clearance, which strongly suggests that [^11^C]QST-0837 irreversibly bound to MAGL *in vivo* and that this was followed by production of ^11^CO_2_.

Based on the results of *in vitro* assays and PET studies using healthy rats, we proposed a compartment model for estimation of the hydrolysis rate (*K*_H_) of [^11^C]QST-0837 *in vivo* (Fig. 5A and B). Since the efflux rate of ^11^CO_2_ was much faster than the *K*_H_,^16^ the *K*_H_ value of [^11^C]QST-0837 could be simply estimated using a mono-exponential fitting of the TAC. Here, because the plasma input function of [^11^C]QST-0837 was almost extinguished 15 min after the injection (Supplementary Fig. 7), exponential fitting on TAC from 15–90 min was permitted. Finally, to evaluate the feasibility of *in vivo* visualization of the pHi gradient based on the *K*_H_ value, we conducted PET experiments with [^11^C]QST-0837 using MCAO rats as a disease model for acute acidic injury resulting from hypoxia (Fig. 5C–H). Several reports using MCAO rats showed that lactate concentrations continued to increase in the insult area until 3–4 h after occlusion.^25,27^ Another report using MCAO piglets showed that lactate levels in the insult area peaked within 2–6 h post-occlusion and then gradually decreased thereafter.^32^ We prepared MCAO rats with different occlusion periods (1, 3–4, or 6 h) and performed PET assessments. These showed that the *K*_H_ value on the ipsilateral side of the brain in MCAO rat decreased, with a peak at 3–4 h of occlusion, which corresponded with the time course of the intracellular concentration of lactate, as described above. Moreover, although there was no correlation between *K*_H_ values and regional differences of CBF in the brains of healthy rat (Supplementary Fig. 6G), the CBF in the ipsilateral brain area of a MCAO rat and immunofluorescence signals reflecting a severely hypoxic area on its brain sections showed strong correlation with the *K*_H_ values (Fig. 5F–H). It is noted that the variation of *K*_H_ value would become larger when maximum SUV was around 0.2 (Fig. 5G). Too much lower CBF (>0.2 SUV) would decrease sensitivity in radioactivity measurements using the PET scanner, although estimation of *K*_H_ is theoretically independent from CBF.

In this study, we could not indicate a direct relationship between *K*_H_ values and pHi in MCAO rats, since there was no way for simultaneous measurement with PET scan for pHi by combining other modalities, such as combination of ^31^P and ^1^H NMRs^33^ and amide proton transfer (APT)–MRI.^34^ However, lactic acidosis has long been associated with hypoxia in brain ischaemia and intracellular lactic accumulation induced by anaerobic glycolysis in severe hypoxic region has also been demonstrated by a number of efforts.^35,36^ Actually, the direct correlation between hypoxia level and pH had been reported under *in vitro* condition.^37^ Therefore, this study has successfully indicated the potential for *in vivo* monitoring of pHi in the brain using PET with [^11^C]QST-0837.

The MCAO model has also been used for APT–MRI, a known pH-weighted imaging technique^26,27,32,38^ that is widely used in clinical studies of hypoxia in cases of stroke and brain tumour.^34,39,40^ However, a significant correlation between APT signals and intracellular lactate levels has not been found.^27,32^ This might be because of technical issues with MRI, which make the technique suitable for morphological imaging but not for functional imaging. Therefore, our proposed PET imaging technique may present a superior alternative to the APT–MRI technique, which suffers from low sensitivity.

In addition to intracellular enzyme activity (as in the case of MAGL), the cytoskeletal component integration, cellular growth and differentiation rates are all highly affected by pHi. The common characteristics of different CNS diseases such as ischaemic stroke, traumatic brain injury, epilepsy, Parkinson’s disease and Alzheimer’s disease are partly induced by decreases in associated enzymes, which show altered activity under acidic conditions.^41^ Furthermore, it is not only ischaemia and tumours that cause intracellular acidification, but also the aging process.^3^ Thus, *in vivo* monitoring of pHi using PET with [^11^C]QST-0837 could be a promising tool for developing new therapeutic approaches against multiple CNS disorders and for understanding the pathological mechanism of the aging process. However, some limitations remain, of which calibration of *K*_H_ against *in vivo* pHi is essential. We are preparing further studies that will combine with MRI techniques for calibration of *K*_H_ values *in vivo.*

## Supplementary Material

fcae172_Supplementary_Data

## Data Availability

Data sharing is not applicable to this article as no new data were created or analysed in this study.
