# Supplementary material for: Potential for in vivo visualization of intracellular pH gradient in the brain using PET imaging
Source: Brain Commun. 2024 May 22;6(3):fcae172. doi: 10.1093/braincomms/fcae172 (PMC11166174; doi:10.1093/braincomms/fcae172)
Supplement: fcae172_Supplementary_Data [file fcae172_supplementary_data.docx]

**Supplementary materials**

**Chemical synthesis**

**General**

^1^H NMR and ^13^C NMR spectra were recorded using a JNM-AL-300 spectrometer (JEOL, Tokyo, Japan) with tetramethylsilane as an internal standard. All chemical shifts (δ) are reported in parts per million (ppm) downfield from the standard. High-resolution mass spectrometry (HRMS) was performed on a JEOL NMS-SX102 spectrometer (JEOL). Column chromatography was performed using a Wakogel C-200 (FUJIFILM Wako Pure Chemical Industries). HPLC was performed on a JASCO HPLC system (JASCO, Tokyo, Japan). Effluent radioactivity was monitored using a NaI (Tl) scintillation detector system. Unless otherwise stated, radioactivity was measured using an IGC-3R Curiemeter (Hitachi Aloka Medical, Tokyo, Japan).

**Synthesis of compound-A**

Compound-A was synthesized according to a reported method,^1,2^ with some modifications as shown in Supplementary Fig. 2A. Briefly, tert-butyl 3-(1-phenyl-1*H*-pyrazol-3-yl)azetidine-1-carboxylate was synthesized by reacting tert-butyl 3-(1*H*-pyrazol-3-yl)azetidine-1-carboxylate (314 mg, 1.4 mmol) with a mixture of phenylboronic acid (343 mg, 2.8 mmol), Cu(OAc)_2_ (383 mg, 2.1 mmol), pyridine (227 μl, 2.8 mmol), and CH_2_Cl_2_ (3 ml) under N_2_. 3-(Azetidin-3-yl)-1-phenyl-1*H*-pyrazole (precursor A) was synthesized via a deprotection reaction of tert-butyl 3-(1-phenyl-1*H*-pyrazol-3-yl)azetidine-1-carboxylate with HCl (4 mol/l 1,4-dioxane, 5.8 ml). 1,1,1,3,3,3-Hexafluoropropan-2-ol (22 μl, 0.2 mmol) in anhydrous CH_2_Cl_2_ (1 ml) was mixed with N,N-diisopropylethylamine (DIPEA) (45 μl, 0.2 mmol) and N-(4-pyridyl)dimethylamine (DMAP) (2.8 mg, 0.02 mmol) and then reacted with triphosgene (21 mg, 0.07 mmol) in CH_2_Cl_2_ (1 ml) at room temperature (r.t.) under N_2_. The reaction mixture was then stirred overnight. 4-(1-Phenyl-1*H*-pyrazol-3-yl)azetidine (50 mg, 0.2 mmol) and DIPEA (120 μl, 0.6 mmol) in CH_2_Cl_2_ (1 ml) were added to the mixture and stirred at r.t. for 5 h. The solution was evaporated, extracted with AcOEt and water, and dried using Na_2_SO_4_. The filtrate was concentrated under vacuum and the obtained crude product was purified using silica gel column chromatography (hexane/ethyl acetate = 6/1) to give compound-A (27 mg, 33%) as a colorless oil. ^1^H NMR (300 MHz, CDCl_3_) 7.90 (d, J = 2.7 Hz, 1H), 7.66 (d, J = 8.4 Hz, 2H), 7.46 (t, J = 7.2, 8.4 Hz, 2H), 7.29 (t, J = 7.8, 7.2 Hz, 1H), 6.41 (d, J = 2.4 Hz, 1H), 5.75–5.63 (m, 2H), 4.55–4.46 (m, 2H), 4.37–4.29 (m, 2H), 4.09–4.01 (m, 1H). HRMS (ESI): calculated m/z for C_16_H_14_O_2_N_3_F_6_ [M+H]^+^: 394.0990; found 394.0999.

The analytical data were identical to the reported data.^1,2^

**Synthesis of tert-butyl 4-(1-phenyl-1*H*-pyrazol-3-yl)piperidine-1-carboxylate**

*(E)*-Tert-butyl 4-(3-(dimethylamino)acryloyl)piperidine-1-carboxylate and tert-butyl 4-(1*H*-pyrazol-3-yl)piperidine-1-carboxylate were synthesized according to a reported method^1^ as shown in Supplementary Fig. 2B. Briefly, tert-butyl 4-acetylpiperidine-1-carboxylate (227 mg, 1 mmol) was reacted with DMF-DMA (1.0 ml, 8 mmol) under reflux for 18 h. The reaction mixture was diluted with toluene (1.0 ml) and evaporated to yield *(E)*-Tert-butyl 4-(3-(dimethylamino)acryloyl)piperidine-1-carboxylate as a brown oil. Subsequently, crude product of *(E)*-Tert-butyl 4-(3-(dimethylamino)acryloyl)piperidine-1-carboxylate was dissolved in EtOH (6 ml), which was reacted with hydrazine hydrate (75 μl, 1.5 mmol) under reflux for 14.5 h. Removal of EtOH yielded tert-butyl 4-(1*H*-pyrazol-3-yl)piperidine-1-carboxylate as a brown oil without further purification. Crude product of tert-butyl 4-(1*H*-pyrazol-3-yl)piperidine-1-carboxylate (251 mg, 1 mmol) was reacted with the mixture containing phenylboronic acid (244 mg, 2 mmol), Cu(OAc)_2_ (272.2 mg, 1.5 mmol), pyridine (161 µl, 2 mmol), and CH_2_Cl_2_ (3 ml) under N_2_ at r.t. for 12 h. The reaction mixture was filtered through celite and the filtrate was concentrated by evaporation. The crude product was purified as a light-yellow solid using silica gel column chromatography (hexane/ethyl acetate = 6/1) to give tert-butyl 4-(1-phenyl-1*H*-pyrazol-3-yl)piperidine-1-carboxylate (272 mg, 83%).^1^H NMR (300 MHz, CDCl_3_) 7.83 (d, J = 2.4 Hz, 1H), 7.65 (d, J = 8.1 Hz, 2H), 7.43 (t, J = 8.1, 8.1 Hz, 2H), 7.27–7.22 (m, 1H), 6.27 (d, J = 2.7 Hz, 1H), 4.26–4.08 (m, 2H), 2.95–2.84 (m, 3H), 2.02–1.97 (m, 2H), 1.75–1.61 (m, 2H), 1.48 (s, 9H).

**Synthesis of** **4-(1-phenyl-1*H*-pyrazol-3-yl)piperidine (precursor B)**

Tert-butyl 4-(1-phenyl-1*H*-pyrazol-3-yl)piperidine-1-carboxylate (271 mg, 0.83 mmol) was treated with HCl (4 mol/l in 1,4-dioxane, 4 ml) and the reaction mixture was stirred at r.t. for 1 h. Removal of the solvents yielded 4-(1-phenyl-1*H*-pyrazol-3-yl)piperidine (precursor B, 140 mg, 75%) as a colorless solid. ^1^H NMR (300 MHz, CDCl_3_) 7.86 (d, J = 2.4 Hz, 1H), 7.65 (d, J = 8.1 Hz, 2H), 7.45 (t, J = 7.8, 7.8 Hz, 2H), 7.32–7.29 (m, 1H), 6.34 (d, J = 2.7 Hz, 1H), 3.76–3.70 (m, 2H), 3.63–3.55 (m, 3H), 3.25–3.10 (m, 2H), 2.35–2.07 (m, 2H). HRMS (ESI): calculated m/z for C_14_H_18_N_3_ [M+H]^+^: 228.1501; found 228.1463.

**Synthesis of compound-P**

A solution of 1,1,1,3,3,3-hexafluoropropan-2-ol (26 μl, 0.2 mmol) in anhydrous CH_2_Cl_2_ (1.5 ml) was reacted with di-2-pyridylcarbonate (52 mg, 0.2 mmol) and DMAP (3.0 mg, 0.02 mmol) at r.t. under N_2_ overnight with stirring. Precursor B (50 mg, 0.22 mmol), DIPEA (115 μl, 0.66 mmol), and CH_2_Cl_2_ (1.0 ml) were added to the mixture and then stirred at r.t. overnight. After the solution had evaporated, the crude product was purified by silica gel column chromatography (hexane/ethyl acetate = 8/1) to give compound-P (21.0 mg, 23%) as a colorless oil. ^1^H NMR (300 MHz, CDCl_3_) 7.84 (d, J = 2.4 Hz, 1H), 7.65 (d, J = 8.1 Hz, 2H), 7.44 (t, J = 7.5, 8.0 Hz, 2H), 7.29–7.24 (m, 1H), 6.27 (d, J = 2.7 Hz, 1H), 5.84–5.72 (m, 1H), 4.26–4.18 (m, 2H), 3.18–3.09 (m, 2H), 3.05–2.97 (m, 1H), 2.11–2.04 (m, 2H), 1.85–1.7 (m, 2H). HRMS (ESI): calculated m/z for C_18_H_18_F_6_N_3_O_2_ [M+H]^+^: 422.1303; found 422.1327.

**Blood sampling and metabolite analysis**

Three Sprague-Dawley rats (male, n = 3, 9–11 weeks old, 326 ± 39 g) were anesthetized with isoflurane (introducing: 5%; keeping: 1.5–2% in air), and a polyethylene catheter (FR2, Imamura, Tokyo, Japan) was inserted into the left femoral artery for blood sampling. The operation for cannulation was performed using an operating microscope (Leica, Wentzler, Germany). Subsequently, a 24-gauge intravenous catheter (Terumo Medical Products, Tokyo, Japan) was inserted into the tail vein of anesthetized rat for bolus injection, and the rat was fixed on an isothermal pad. A bolus of [^11^C]QST-0837 (51 ± 5 MBq, 1 ml) was injected at a flow rate of 0.5 ml/min using a syringe pump (PHD2000, Harvard Apparatus, MA, USA) via a catheter in the tail vein. Arterial blood was manually collected via catheter of tail vein into the microtube at 20 and 40 s (0.05 ml); 60 s (0.1 ml); 80, 100, 110, 120, 130, and 150 s and 3 and 4 min (0.05 ml); 5 min (0.2 ml); 10 min (0.08 ml); 15 min (0.2 ml); 30 min (0.3 ml); 60 min (0.4 ml), and 90 min (0.5 ml) after the injection. The blood samples were weighted and centrifuged at 15,000 *g* at 4°C to separate the plasma. The radioactivity of the whole blood and plasma was determined using a 2480 Wizard auto-gamma scintillation counter (Perkin-Elmer, Waltham, MA, USA) and was corrected for decay. Metabolite analysis was performed as described previously.^4^ Briefly, plasma samples obtained 1, 5, 15, 30, 60, and 90 min after injection were deproteinized with equivalent volumes of acetonitrile. An aliquot of the supernatant from the plasma was injected into an HPLC system with a radiation detector, and analyzed using a Capcell Pack C18 column (4.6 mm i.d. × 250 mm, Shiseido, Tokyo, Japan) with MeCN/H_2_O (8/2, v/v, 0.1% Et_3_N) at 1.2 ml/min.

**Supplementary Fig. 1**

**
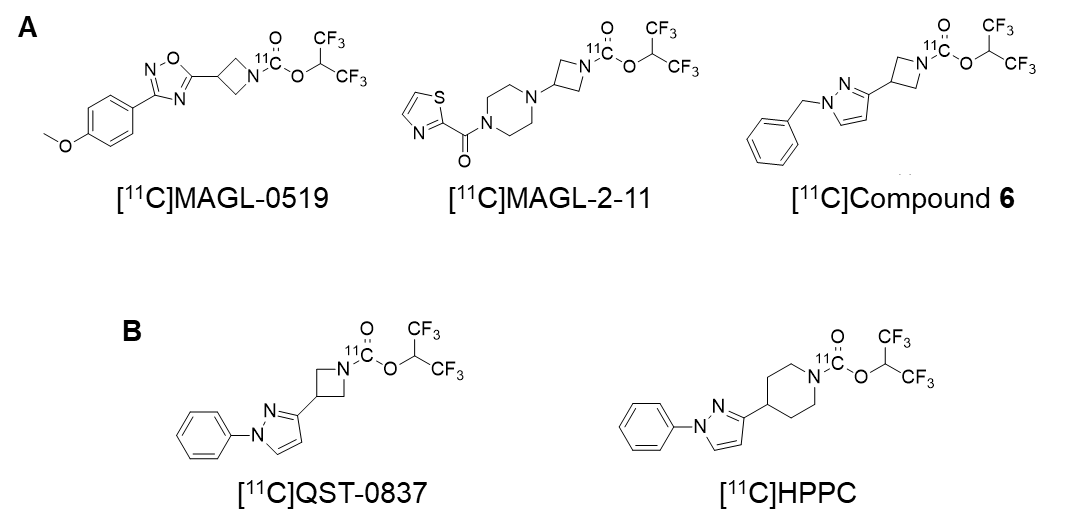
**

**Supplementary Fig 1. PET probes consisted in covalent inhibitors for monoacylglycerol lipase.** (**A**) Previous PET probes containing azetidine carbamate skeleton. (**B**) Novel PET probes containing azetidine carbamate ([^11^C]QST-0837) or piperidine carbamate ([^11^C]HPPC) developed in this study.

**Supplementary Fig. 2**

**
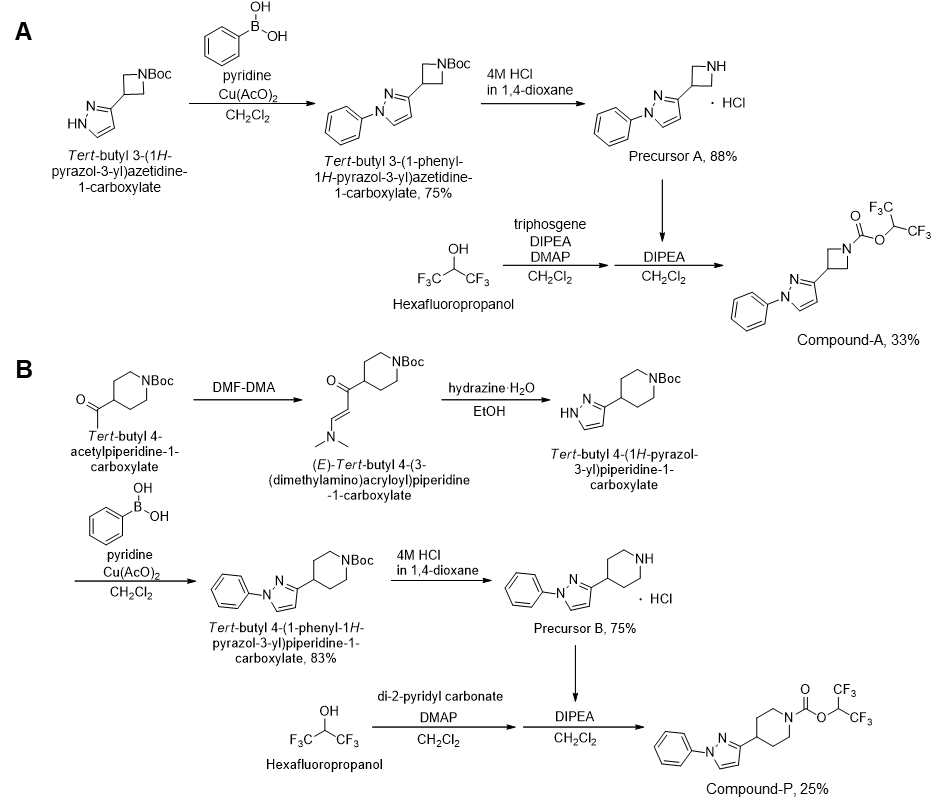
**

**Supplementary Fig 2. Chemical syntheses of compound-A and compound-P.** (**A**) Scheme of chemical synthesis of compound-A. Compound-A was synthesized by reacting with precursor A produced starting from tert-butyl 3-(1*H*-pyrazol-3-yl)azetidine-1-calboxylate and 1,1,1,3,3,3-hexafluoropropan-2-ol. (**B**) Scheme of chemical synthesis of compound-P. Compound-P was synthesized by reacting with precursor B derived from tert-butyl 4-(1-phenyl-1*H*-pyrazol-3-yl)piperidine-1-carboxylate and 1,1,1,3,3,3-hexafluoropropan-2-ol. Abbreviations: DIPEA, N,N-diisopropylethylamine; DMAP, N-(4-pyridyl)dimethylamine.

**Supplementary Fig. 3**

**
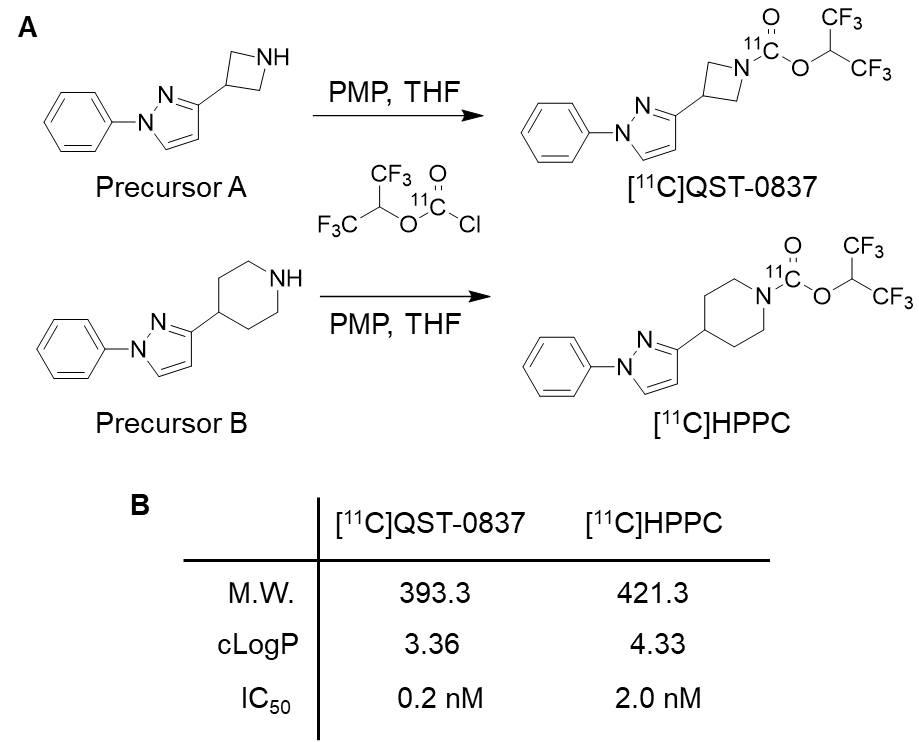
**

**Supplementary Fig 3. Radiosynthesis of [^11^C]QST-0837 and [^11^C]HPPC.** (**A**) [^11^C]QST-0837 and [^11^C]HPPC were synthesized by reacting respective precursors (A for [^11^C]QST-0837, B for [^11^C]HPPC) and [^11^C]chloroformate in a solution containing 1,2,2,6,6-pentamethylpiperidine (PMP) and tetrahydrofuran (THF). (**B**) General profiles (molecular weight, lipophilicity, and IC_50_ for MAGL) of [^11^C]QST-0837 and [^11^C]HPPC.

**Supplementary Fig. 4**

**
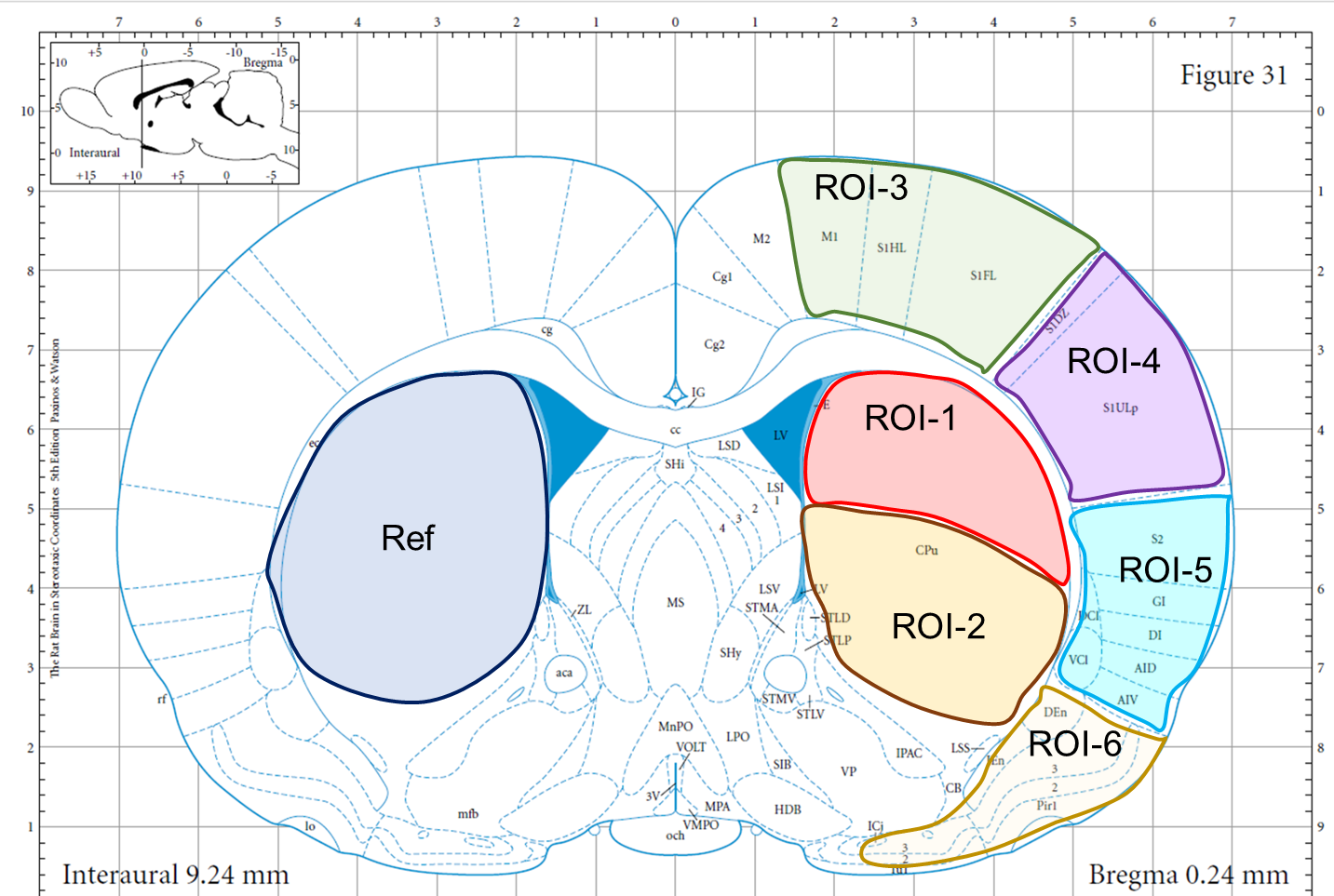
**

**Supplementary Fig 4. Regions of interest (ROIs) in images of immunofluorescence staining corresponding to PET images of MCAO rat.^3^** The ROIs were manually drawn on the top of striatum (ROI-1), bottom of striatum (ROI-2), primary somatosensory cortex (ROI-3), secondary somatosensory cortex (ROI-4), insular cortex (ROI-5), and piriform cortex (ROI-6) of ipsilateral hemisphere. The ROI on the striatum of contralateral hemisphere is described as the reference.

**Supplementary Fig. 5**

**B**

**A**

**Supplementary Fig 5. Blocking studies for PET with [^11^C]QST-0837 and [^11^C]HPPC.** (**A**) TACs of [^11^C]QST-0837 in the whole brain of rat administrated without (baseline, n = 4) or with self-blocking or JW642 of different doses [1 (n = 3) and 3 mg/kg (n = 2)]. The radioactive uptake in the brain was significant decreased by self-blocking or JW642 treatment (P < 0.05). The blockade by JW642 made more reduction than that by self-blocking. These differences might be caused by overflows from peripheral organs by self-blocking. There were no significant differences between different doses of both blockers (P > 0.05). (**B**) TACs of [^11^C]HPPC in the whole brain of rat administrated without (baseline, n = 4) or with HPPC or JW642 of different doses [1 (n = 3) and 3 mg/kg (n = 2)]. Although intensive increments of radioactive uptake in initial phase were seen in subjects treated with JW642, radioactivity in the brain of rat treated with blocking agent significantly subsequently decreased to 0.5 SUV. There were no significant differences between different doses of both blockers (P > 0.05). Statistically significant differences were calculated by one-way ANOVA with Tukey’s post hoc test.

**Supplementary Fig. 6**

**
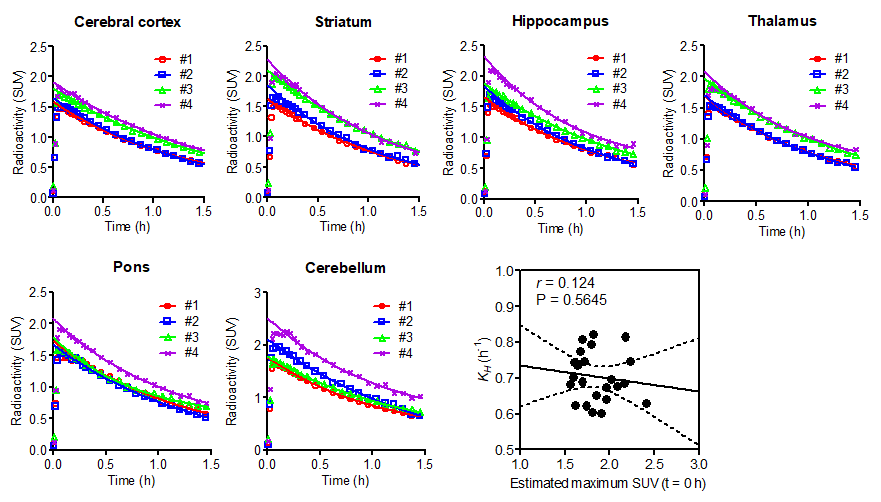
**

**G**

**F**

**E**

**D**

**C**

**B**

**A**

**Supplementary Fig 6. Time-activity curves (TACs) of [^11^C]QST-0837 and fitting curves in individual rats.** The volumes of interest (VOIs) for estimation of TACs in brain regions were drawn in the cerebral cortex (**A**), striatum (**B**), hippocampus (**C**), thalamus (**D**), pons (**E**), and cerebellum (**F**). The clearance rate of radioactivity (K_H_) was estimated by a one-exponential fitting on the TACs from 15 to 90 min. (**G**) Relationship between K_H_ value and estimated maximum radioactivity (SUV). Estimated maximum SUV was calculated by exponential equation when t was 0. There were no correlations between K_H_ value and estimated maximum SUV (*r* = 0.124). Relationship test was conducted by a linear regression.

**Supplementary Fig. 7**

**B**

**A**

**Supplementary Fig 7. Plasma input curve of [^11^C]QST-0837 in healthy rats (n = 3). (A)** The composition of unchanged form of [^11^C]QST-0837 in plasma during a PET scan. (**B**) Time-activity curves of [^11^C]QST-0837 in the whole blood (red circles), plasma (blue circles), and metabolite-corrected input function. Radioactivity was expressed as SUV.

**References**

1. Butler CR, Beck EM, Harris A, et al. Azetidine and Piperidine Carbamates as Efficient, Covalent Inhibitors of Monoacylglycerol Lipase. *J Med Chem.* 2017;60:9860-9873.

2. Jones A, Kemp M, Stockley M, Gibson K, Whitlock G. 1-CYANO-PYRROLIDINE COMPOUNDS AS USP30 INHIBITORS. 2016;WO 2016/156816 A1.

3. Paxinos G, Watoson C. The rat brain in stereotaxic coordinates. 5^th^ edition. Elsevier Academic Press; 2005.

4. Yamasaki T, Fujinaga M, Yui J, et al. Noninvasive quantification of metabotropic glutamate receptor type 1 with [11C]ITDM: a small-animal PET study. *J Cereb Blood Flow Metab.* 2014;34:606–612.
